# Supplementary material for: Suggested experimental design and computational modeling to infer single-cell lipid dynamics from a single destructive measurement
Source: iScience. 2026 Mar 25;29(4):115474. doi: 10.1016/j.isci.2026.115474 (PMC13091468; doi:10.1016/j.isci.2026.115474)
Supplement: Document S1. Figures S1−S5 and Tables S1 and S2 [file mmc1.pdf]

iScience, Volume *29*

Supplemental information

Suggested experimental design and computational modeling to infer single-cell lipid dynamics from a single destructive measurement

Paul Jonas Jost, Daniel Weindl, Klaus Wunderling, Christoph Thiele, and Jan Hasenauer

Supplementary Figures and Tables

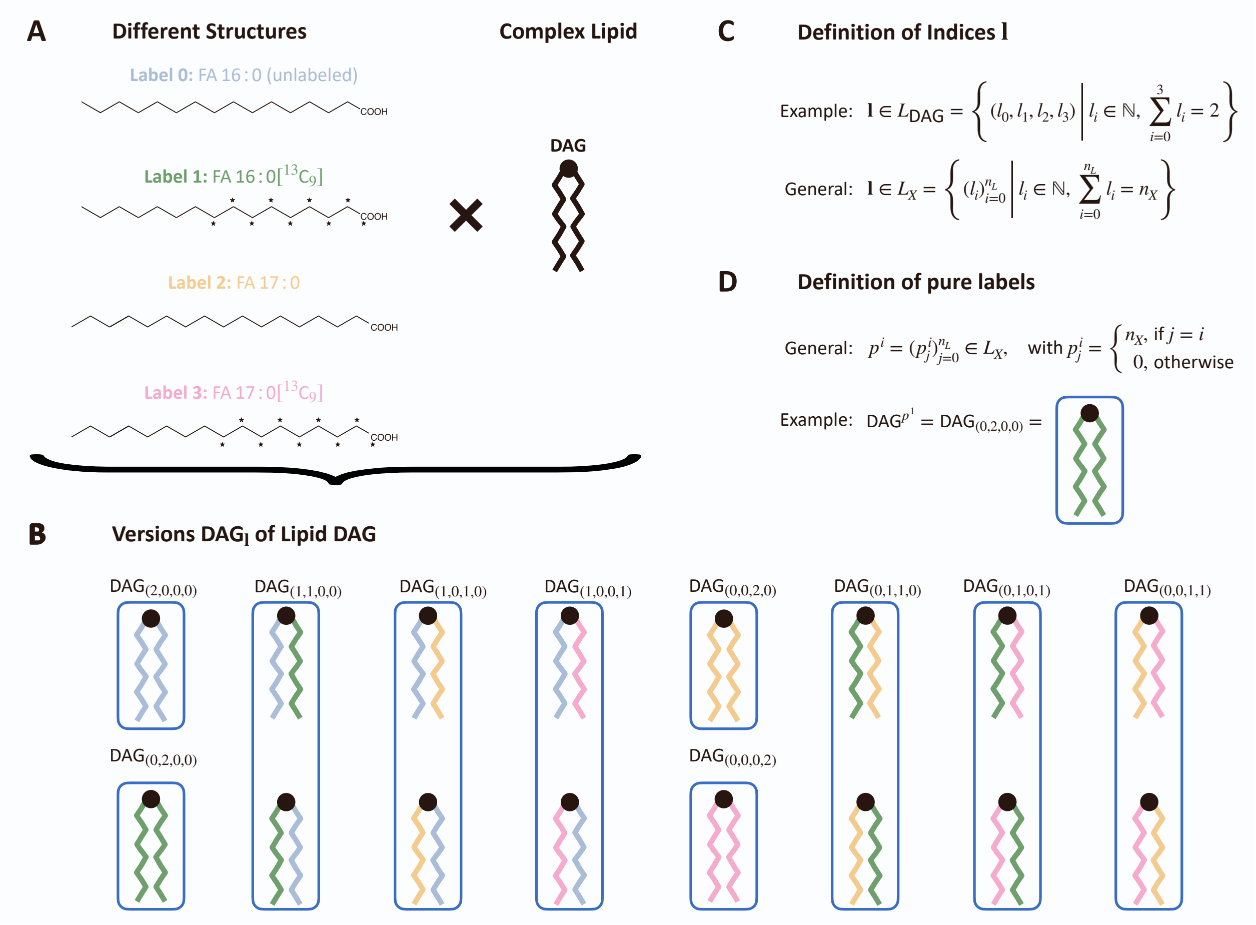

Figure S1: **Visual explanation of labeling and notation, related to Figure 3.** (A) We use different - but chemically similar - alkyne-fatty acids as metabolic labels. Therefore, any complex lipid (e.g., can now have multiple versions of itself depending on the label combination. (B) In the case of DAG, with three labels and one unlabeled, this results in 16 different versions. As we assume independence of the position, some versions are deemed equivalent in our model (blue boxes). Thus, the complexity of DAG is reduced to ten versions. (C) Definition of the index notation. Each entry i in the vector index represents the number of labels i in the specific variant of the DAG. They add up to the complex lipid's number of lipid binding sites. (D) For ease of notation, we also introduce the purely labeled lipids. DAG^{p^1} represents the variant of DAG that has label 1 at each binding site.

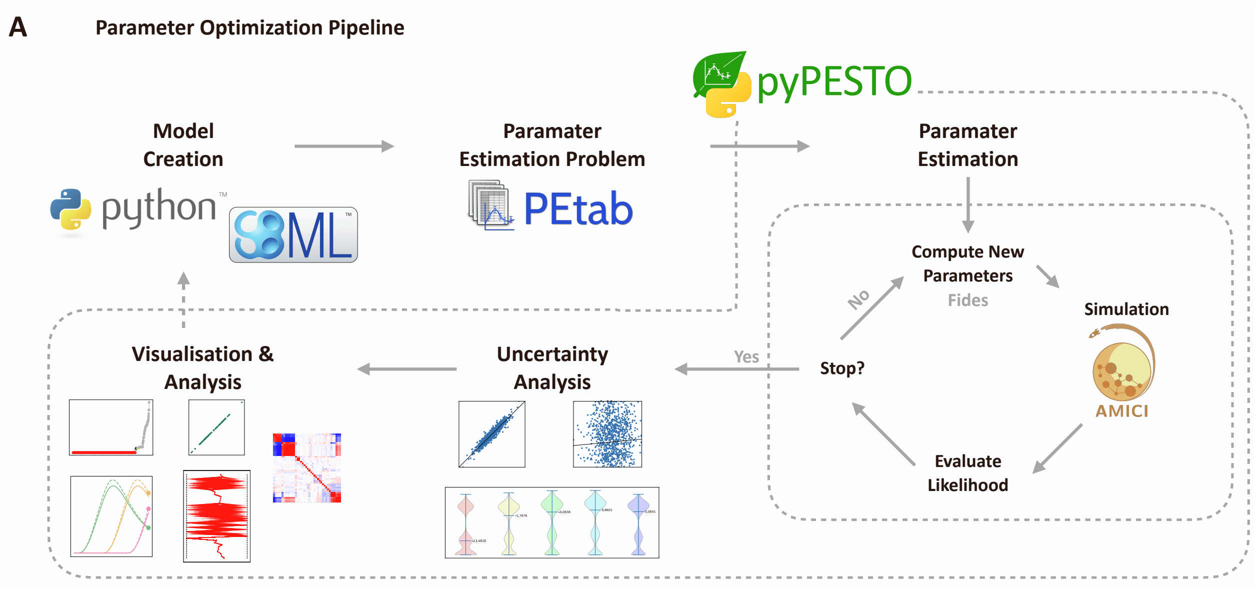

Figure S2: **Parameter Estimation Pipeline, related to STAR Methods.** (A) The pipeline starts by creating an SBML model using a self-implemented rule-based model written in Python. Using the SBML model and the synthetically created parameters, PEtab problems for each cell are generated. Subsequently, pyPESTO is used for parameter estimation, uncertainty analysis, and visualization. Parameter estimation is a cycle of simulation, likelihood evaluation, and new parameter calculation. For estimation, the Fides optimizer is interfaced, and AMICI is used for simulation.

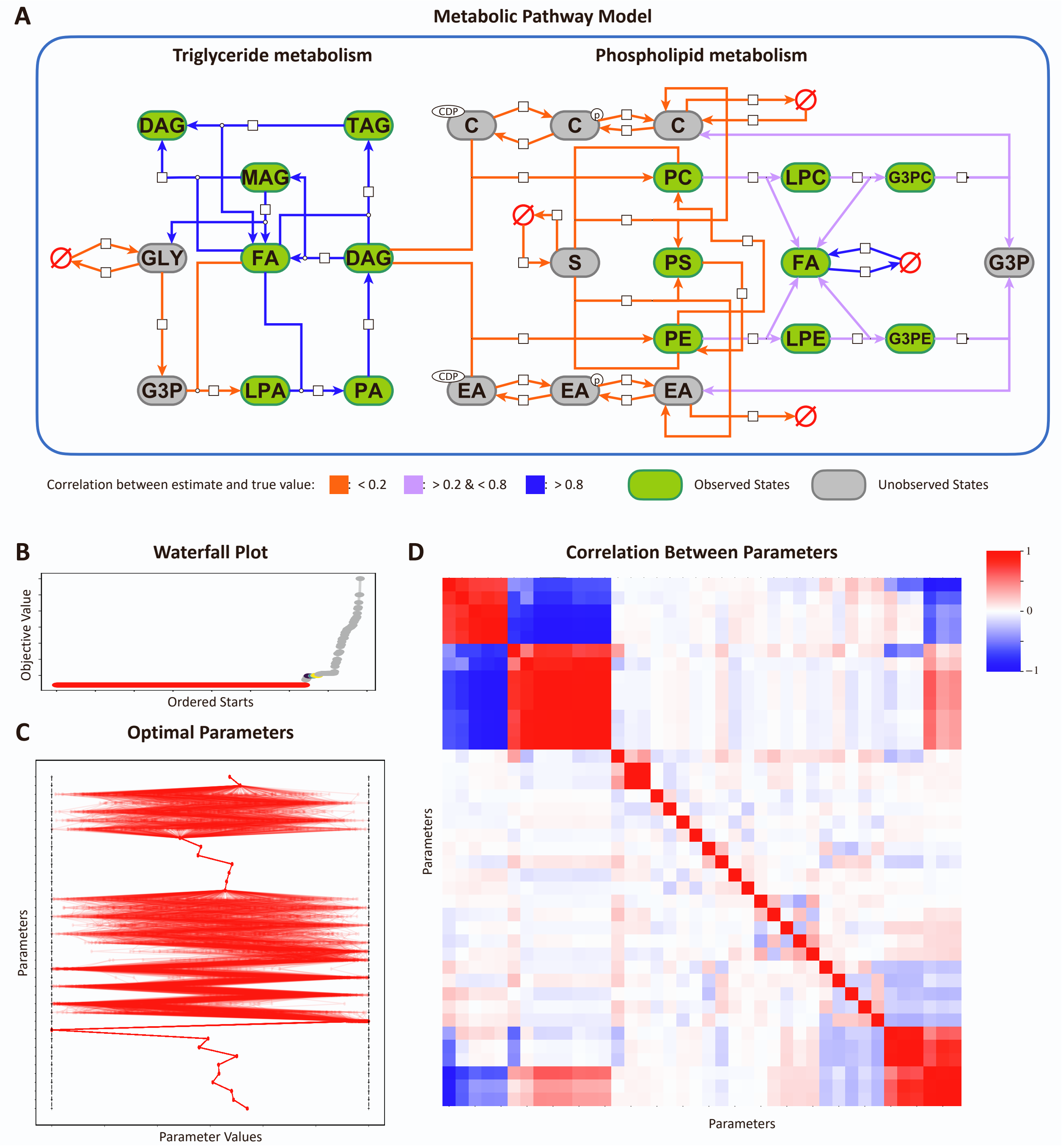

Figure S3: **Parameter uncertainties can be linked to unobservability, related to Figure 5.** (A) A graphical representation of the pathway model from Figure 3. Reactions are additionally colored by the correlation between the true and estimated parameter value of the corresponding parameter. (B) Waterfall plot of one multi-start local optimization. The final objective values of each start are sorted. Starts with the same color (except grey) correspond to almost equal final values. (C) Final parameter values of the best 400 starts plotted between the parameter bounds. All 400 starts had almost equal objective function values. (D) Correlation between parameters across the best 400 starts.

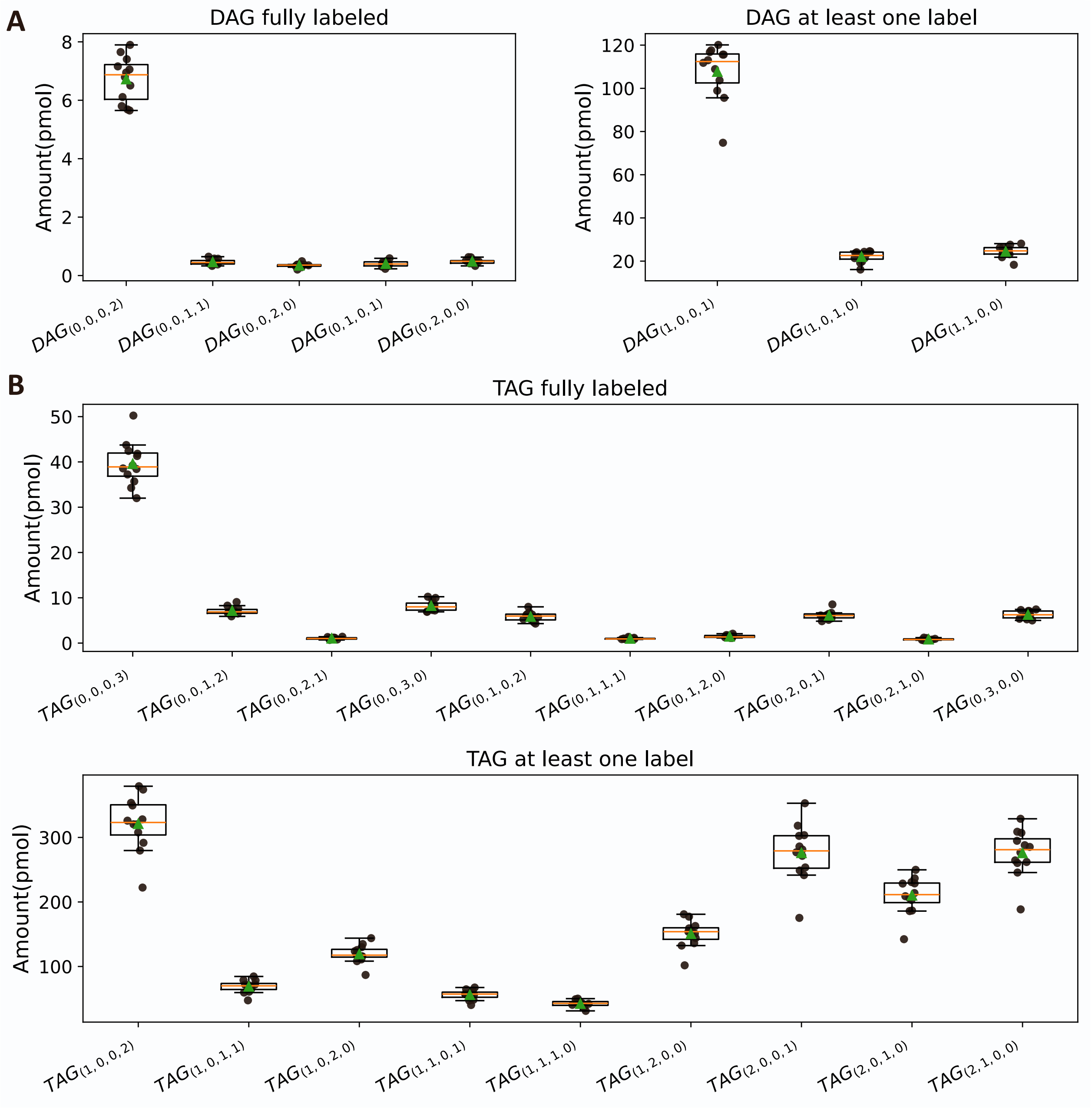

Figure S4: **Feasibility of experimental setup in bulk-cell context - grouped by labels, related to Figure 2.** Data from a bulk-lipidomics with an experimental setup as described in Figure 2. All boxplots depict first and third quartile, median, whiskers extending to $1.5 \cdot$ IQR, as well as extreme points outside the whiskers. (A) DAG measurements, (B) TAG measurements. Measurements are in pmol. The indices are the number of fatty acids that function as “no label”, “label 1”, “label 2”, and “label 3”. Figure S1contains a detailed example for this annotation. They are separated based on whether they have only labeled fatty acyls (“fully labeled”) or not (“at least one label”).

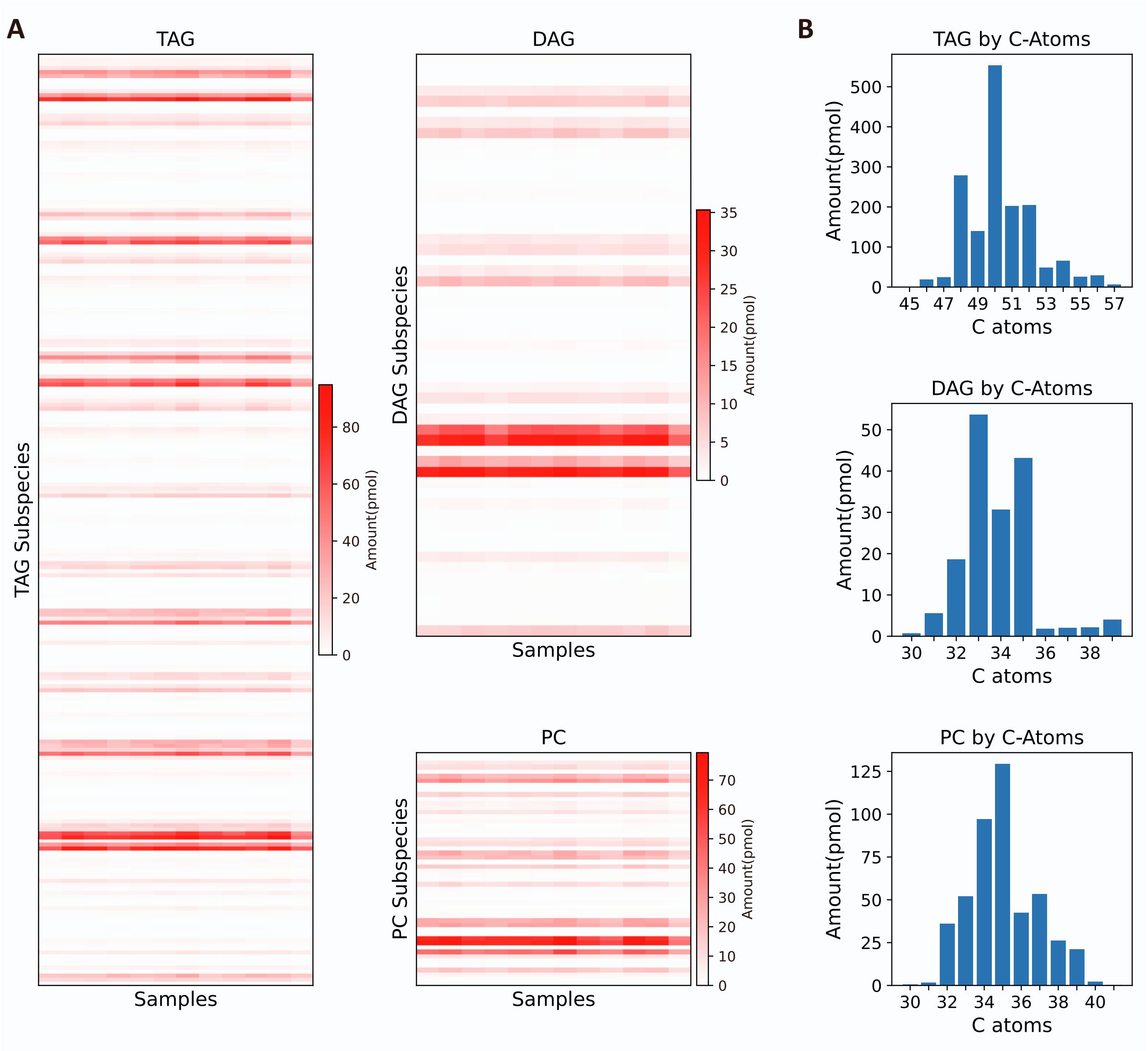

Figure S5: **Feasibility of experimental setup in bulk-cell context - lipid distribution, related to Figure 2.** (A) A heatmap looking into bulk-lipidomics measurement using the experimental setup in Figure 2. Each row corresponds to a measured lipid of the corresponding class (TAG, DAG, PC), each column corresponds to a separate sample. The coloring is according to the measured amount in pmol. (B) A distribution plot, grouping lipids from their respective lipid class based on their number of carbon atoms in the fatty acyls.

Table S1: **List of the state variables in the mathematical model and their corresponding abbreviations, related to STAR Methods.**

Name	Description
FA	Fatty acids
GLY	Glycerol
G3P	Glycerol-3-phosphates
LPA	Lysophosphatidic acids
PA	Phosphatidic acids
MAG	Monoacylglycerols
DAG	Diacylglycerols
TAG	Triacylglycerols
PE	Phosphatidylethanolamines
PC	Phosphatidylcholines
PS	Phosphatidylserines
EA	Ethanolamine
EA_{CDP}	cytidine diphosphate (CDP) -ethanolamine
EA_{PH}	Phosphoethanolamine
C	Choline
C_{CDP}	CDP-Choline
C_{PH}	Phosphocholine
S	Serine
LPE	Lysophosphatidylethanolamines
LPC	Lysophosphatidylcholines
G3PE	Glycero-phosphoethanolamines
G3PC	Glycero-phosphocholines

Table S2: **Reactions in the mathematical model, related to STAR Methods.** The first column indicates the rules used, i.e., the reactions of the base model without labels. In the second column, the corresponding rate laws - using the reactants in their base form - are listed. The third column shows how the number of reactions can be calculated through the metabolites participating in the reaction and their corresponding amount of labeling sites. The last column shows an example of the case of three labels. n_L is the number of distinct labels used.

Rule	Rate Law	Number of Reactions	
		Formula	Example $n_L = 3$
$\emptyset \longleftrightarrow FA$	$k_{28} - k_{31}[FA]$	$n_L + 1$	4
$\emptyset \longleftrightarrow GLY$	$k_{27} - k_{32}[GLY]$	1	1
$GLY \longleftrightarrow G3P$	$k_1[GLY] - k_{35}[G3P]$	1	1
$G3P + FA \longrightarrow LPA$	$k_2[G3P][FA]$	$n_L + 1$	4
$LPA + FA \longrightarrow PA$	$k_3[LPA][FA]$	$(n_L + 1)^2$	16
$PA \longrightarrow DAG$	$k_4[PA]$	$\binom{2+n_L}{n_L}$	10
$DAG + FA \longleftrightarrow TAG$	$k_5[DAG][FA] - k_{24}[TAG]$	$\binom{2+n_L}{n_L}(n_L + 1)$	40
$DAG \longleftrightarrow MAG + FA$	$k_{22}[DAG] - k_{23}[MAG][FA]$	$(n_L + 1)^2$	16
$MAG \longrightarrow GLY + FA$	$k_{25}[MAG]$	$n_L + 1$	4
$\emptyset \longleftrightarrow C$	$k_{36} - k_{37}[C]$	1	1
$C \longleftrightarrow C_{PH}$	$k_6[C] - k_{33}[C_{PH}]$	1	1
$C_{PH} \longleftrightarrow C_{CDP}$	$k_7[C_{PH}] - k_{38}[C_{CDP}]$	1	1
$EA \longrightarrow \emptyset$	k_{40}	1	1
$EA \longleftrightarrow EA_{PH}$	$k_8[EA] - k_{34}[EA_{PH}]$	1	1
$EA_{PH} \longleftrightarrow EA_{CDP}$	$k_9[EA_{PH}] - k_{39}[EA_{CDP}]$	1	1
$\emptyset \longleftrightarrow S$	$k_{26} - k_{29}[S]$	1	1
$DAG + EA \longrightarrow PE$	$k_{10}[DAG][EA_{CDP}]$	$\binom{2+n_L}{n_L}$	10
$DAG + C \longrightarrow PC$	$k_{11}[DAG][C_{CDP}]$	$\binom{2+n_L}{n_L}$	10
$PC + S \longrightarrow PS + C$	$k_{12}[PC][S]$	$\binom{2+n_L}{n_L}$	10
$PE + S \longrightarrow PS + EA$	$k_{13}[PE][S]$	$\binom{2+n_L}{n_L}$	10
$PS \longrightarrow PE$	$k_{14}[PS]$	$\binom{2+n_L}{n_L}$	10
$PE \longrightarrow PC$	$k_{15}[PE]$	$\binom{2+n_L}{n_L}$	10
$PC \longrightarrow \emptyset$	$k_{30}[PC]$	$(n_L + 1)^2$	16
$PC \longrightarrow LPC + FA$	$k_{16}[PC]$	$(n_L + 1)^2$	16
$LPC \longrightarrow G3PC + FA$	$k_{17}[LPC]$	$n_L + 1$	4
$G3PC \longrightarrow G3P + C$	$k_{18}[G3PC]$	1	1
$PE \longrightarrow LPE + FA$	$k_{19}[PE]$	$(n_L + 1)^2$	16
$LPE \longrightarrow G3PE + FA$	$k_{20}[LPE]$	$n_L + 1$	4
$G3PE \longrightarrow G3P + EA$	$k_{21}[G3PE]$	1	1

Data S1/Methods S1: Mathematical Proof and Bulk Experiment

Proof of Time Shift

In this section, we will mathematically prove that under certain assumptions, a model that incorporates different labels at different time points allows us to obtain a pseudo-time trajectory.

Assumptions

We consider processes whose dynamics are governed by reactions with fluxes following the law of mass action kinetics. We note that Michaelis-Menten kinetics can be considered as well. While this case is not considered in this proof, we will outline how this can be derived afterward. As the reaction parameters are not the focus of this section, we shall slightly deviate from the notation in "Mathematical Models" in the methods section.

In general, we define a metabolic reaction network as a number of reactions R_i between metabolites, i.e.

$$R_i : \sum_{j=1}^{|\mathcal{X}|} s_{i,j}^- X_j \longrightarrow \sum_{j=1}^{|\mathcal{X}|} s_{i,j}^+ X_j, \quad i = 1, \ldots, n_R, \tag{1}$$

in which $n_R \in \mathbb{N}$ is the number of reactions, $s_{i,j}^-$, $s_{i,j}^+ \in \mathbb{N}$ indicate the stoichiometry of reactants and products, respectively, $\mathcal{X}$ denotes the species considered in the reaction network and $|\mathcal{X}|$ the number of species considered. We only consider reactions up to the second order, as reactions of any higher order can either be written as chains of reactions up to the second order or can be neglected[1]. Therefore, our reaction network is simplified to

$$R_i : \; aX + bY \longrightarrow \textit{products}, \quad a, b \in \{0, 1\}, \; X, Y \in \mathcal{X}, \; i = 1, \ldots, n_R, \tag{2}$$

Given a reaction network, its dynamics can be modeled with a system of ordinary differential equations with the following right-hand side,

$$\frac{dx}{dt} = f(x(t,\theta), \theta) = Sv(x(t,\theta), \theta), \qquad x(t_0) = x_0, \tag{3}$$

in which $x(t, \theta) \in \mathbb{R}^n$ denotes the vector of state variables corresponding to different species, S is the stoichiometric matrix, $v \in \mathbb{R}^{n_R}$ is the flux vector of the reactions and θ are the model parameters associated with the reaction fluxes. We shall denote a specific species with $X \in \mathcal{X}$ or $Y \in \mathcal{X}$ and write $\frac{dX}{dt}$ for its corresponding differential equation. Additionally, we assume that the system has a steady state, i.e.

$$\exists\, \bar{x}(\theta) \in \mathbb{R}^n \text{ s.t. } f(\bar{x}(\theta), t) = 0 \;\forall t \geq 0$$

We extend this model by incorporating a labeling strategy (Figure S1). With n_L, we denote the number of different labels L_i, $i = 0, \ldots, n_L$, in which L_0 represents the absence of a label. For each species $X \in \mathcal{X}$, we denote the number of labeling sites X has with n_X. We define the species X with a specific combination of labels $\mathfrak{l}$ as $X^{\mathfrak{l}}$, in which

$$\mathfrak{l} \in L_X = \left\{ \mathfrak{l} \,\middle|\, \mathfrak{l} = (l_i)_{i=0}^{n_L} \in \mathbb{N}_0^{n_L+1}, \sum_{i=0}^{n_L} l_i = n_X \right\}, \tag{4}$$

is a multi-index from the set of possible multi indices L_X for species X. Here, l_i represents the number of labels L_i the species $X^{\mathfrak{l}}$ has. This notation has an implicit dependency on a species X, but as the multi-index is always used in combination with X, the context resolves this dependency.

Of specific interest are pure combinations that only consist of one label. For example, the species X^{p^i} that only has labels of the kind L_i, i.e.

$$p^i = (p^i_j)_{j=0}^{n_L} \in L_X, \quad \text{with } p^i_j = \begin{cases} n_X, & \text{if } j = i \\ 0, & \text{otherwise} \end{cases} . \tag{5}$$

Given this setup, we assume the reactions to be independent of the labels:

Assumption 1. *Given a labeling defined in* (4) *and a reaction network, we combine them into a labeled reaction network in assuming the following*

1. *For each reaction $R_i :\ aX + bY \longrightarrow$ products, any labeling of X or Y can trigger reaction R_i, i.e. for all $\mathbf{l} \in L_X$, $\mathbf{l}' \in L_Y$, the reaction*

$$R_i^{\mathbf{l},\mathbf{l}'} :\ aX^{\mathbf{l}} + bY^{\mathbf{l}'} \longrightarrow \text{products}$$

   is a valid reaction of the labeled metabolic reaction network.

2. *The reaction rates are independent of the labels, i.e. given two reactions $R_i^{\mathbf{l},\mathbf{l}'}$, $R_i^{\tilde{\mathbf{l}},\tilde{\mathbf{l}}'}$, if the concentrations of $X^{\mathbf{l}}$ and $X^{\tilde{\mathbf{l}}}$ as well as $Y^{\mathbf{l}'}$ and $Y^{\tilde{\mathbf{l}}'}$ are equal, then the fluxes of both reactions are equal.*

In summary, Assumption 1.1 implies that a reaction can occur with any labeled version of its reactants. The labels on the products are dependent on the reactants. The second part assumes that reactions that only differ in the labeled versions of their reactants have the same reaction rates.

As per our experimental setup, the influx of the labels must be time-shifted versions of one another, except for label L_0.

Assumption 2. *Given a number n_L of different labels L_i, $i = 0, \ldots, n_L$ that satisfy Assumption 1, we assume that for all reactions R_{L_i} that represent an influx of label L_i, $i = 0, \ldots, n_L$ into the system, there is a corresponding reaction R_{L_j}, $j = 0, \ldots, n_L$ such that*

1. *The fluxes of those reactions can be described as time-shifts of one another, i.e.*
   $\nu_{R_{L_i}}(t) = \nu_{R_{L_j}}(t + T_{i,j})$, $T_{i,j} \in \mathbb{R}$, for all $1 \leq i, j \leq n_L$, in which $T_{i,j}$ is the time-shift between labels L_i and L_j.

2. *The overall influx of all labels combined is constant, i.e., $\sum_{i=0}^{n_L} \nu_{R_{L_i}}(t) = C$, $C \in \mathbb{R}$.*

Here, we assumed that the influxes of labels are time-shifted versions of one another and that the total influx is constant. Assumption 2.1 will ensure that the dynamics of the whole system are time-shifted of one another. Assumption 2.2 guarantees that the overall system remains in a steady state throughout the time course. An example would be the uptake of fatty acids in a cell, where we wash the cells at specific times and reapply a medium that only differs in the fatty acid content from the previous one.

Theorem and Proof

For the considered process, the following holds:

Theorem 1. *Given metabolic reaction network* (3) *with steady-state $\bar{x} \in \mathbb{R}^n$ and that Assumptions 1 and 2 hold, the following is ensured:*

1. *Given that we start in a steady state with only unlabeled metabolites, the sum over all labels of lipids is in a steady state, i.e.*

$$\forall X \in \mathcal{X} :\ \frac{d}{dt} \sum_{\mathbf{l} \in L_X} X^{\mathbf{l}} = 0$$

2. *For all $X \in \mathcal{X}$, the biochemical species that contain only one type of label are time-shifted versions of one another, i.e.*

$$\forall X \in \mathcal{X} \, \forall 1 \le i, j \le n_L : X^{p^i}(t) = X^{p^j}(t + T_{i,j}),$$

in which $T_{i,j} \in \mathbb{R}$ is defined in Assumption 2.

Proof. 1. We proof the first statement with a divide and conquer approach. We note that the ODE system stems from reactions, and thus, the differential equation for each species can be represented as a sum of reactions in which this species is involved. Additionally, we only consider reactions of up to the second order that follow mass action kinetics. Thus, it suffices to prove our point for each of those reactions individually. We will show that summing over the label combinations will yield the same ODE as the unlabeled reaction network for all three reactions. We use Assumption 1.1, by which reactions exist for all $\mathbf{l} \in L_X$ or none. We also make use of Assumption 2.2

(a) Zeroth order: By Assumption 2.2, the sum over any influx is constant. Thus, we can write straightforward

$$R_1 : \emptyset \longrightarrow X \;\Rightarrow\; \nu_1 = k_1$$

$$\sum_{\mathbf{l} \in L_X} \frac{dX^{\mathbf{l}}}{dt} = \frac{d}{dt} \sum_{\mathbf{l} \in L_X} X^{\mathbf{l}} = \sum_{\mathbf{l} \in L_X} k_1 = C \tag{6}$$

(b) First order:

$$R_2 : X \longrightarrow Y \;\Rightarrow\; \nu_2 = k_2 X$$

We first take a look at the product site

$$\frac{d}{dt} \sum_{\mathbf{l} \in L_X} X^{\mathbf{l}} = \sum_{\mathbf{l} \in L_X} -k_2 X^{\mathbf{l}} = -k_2 \frac{d}{dt} \sum_{\mathbf{l} \in L_X} X^{\mathbf{l}} \tag{7}$$

Now we use the fact that $\sum\limits_{\mathbf{l} \in L_X} \dfrac{dX^{\mathbf{l}}}{dt} + \sum\limits_{\mathbf{l} \in L_Y} \dfrac{dY_{\mathbf{l}}}{dt} = 0$, and thus, we can follow the same for the product(s) Y.

(c) Second order:

$$R_3 : X + Y \longrightarrow \text{products} \;\Rightarrow\; \nu_3 = k_3 XY$$

Without loss of generality, we look at X

$$\frac{d}{dt} \sum_{\mathbf{l} \in L_X} X^{\mathbf{l}} = \sum_{\mathbf{l} \in L_X} -k_3 X^{\mathbf{l}} \sum_{\mathbf{l}' \in L_Y} Y_{\mathbf{l}'} = -k_3 \sum_{\mathbf{l} \in L_X} X^{\mathbf{l}} \sum_{\mathbf{l}' \in L_Y} Y_{\mathbf{l}'} \tag{8}$$

Following the same argumentation as in the first order, we can deduce the same for Y and the products.

Now we can combine those, and by defining $\mathbf{X} := \sum\limits_{\mathbf{l} \in L_X} X^{\mathbf{l}}$, we can see that the differential equations agree with the original unlabeled network. Thus, a steady state of the sum is also a steady state of the unlabeled metabolic network and will, therefore, stay in a steady state.

2. In the following, we prove the second part of the theorem by rewriting reactions and species by summing up all species with a specific number of labels L_i. This creates a system of equations for label L_i, $i > 0$. Since i is arbitrary, we can conclude, in combination with Assumption 2.1, that these systems are time-shifted versions of one another. The purely labeled species are then only a special case.

The amount of species X that has exactly m times label i is defined by:

$$S(i, X, m)(t) = \sum_{\mathbf{l} \in L_X | l_i = m} m X^{\mathbf{l}}(t).$$

We can retrieve $\frac{dS(i,X,1)}{dt}$ by rewriting reactions in terms of exchanging labels. Given a reaction $Y \longleftrightarrow X + Z$ with flux kY. Without loss of generality, let us assume $n_Y = 2, n_X = n_Z = 1$, in which n_Y, n_X, n_Z are taken from (4). Let us now look at $S(i, Y, m)$ for differing values of m.

(a) $m = 0, m = 2$
Y^1 without a label L_i ($l_i = 0$) cannot pass on a label L_i to X and will always end up in $S(i, X, 0)$. Therefore, we end up with

$$\frac{dS(i,X,0)}{dt} = kS(i,Y,0) + R, \tag{9}$$

in which R indicates the terms of $S(i, Y, 1)$ and $S(i, Y, 2)$ that are considered in the next cases.

(b) $m = 2$
$S(i, Y, 2)$ only has label L_i, therefore it will always pass on L_i in an exchange. Thus

$$\frac{dS(i,X,1)}{dt} = kS(i,Y,2) + R. \tag{10}$$

(c) $m = 1$
Given that we have two labels in Y^1 of which exactly one is label i, the probability of passing on the label to X is 50%. Therefore, half of the fluxes from $S(i, Y, 1)$ go to $S(i, X, 1)$ and the other half to $S(i, X, 0)$. We thus can extend equations (9) and (10)

$$\frac{dS(i,X,0)}{dt} = kS(i,Y,0) + 0.5kS(i,Y,1)$$
$$\frac{dS(i,X,1)}{dt} = kS(i,Y,2) + 0.5kS(i,Y,1).$$

Any other reaction will follow the same reasoning, slightly altered depending on n_X and n_Y.

Since we can describe the system $(S(i, X, m))_{X\in\mathcal{X},m\leq n_X}$ independently of other labels, the label i is interchangeable. As all $S(i, X, m)$ with $m > 1$ are zero before the influx of label i, $\forall t < T_i, m \geq 1$: $S(i, X, m) = 0$ and in those cases $S(i, X, 0)$ is equal for all i, and we additionally know from Assumption 2.1, that the influxes are time-shifted versions of one another, we conclude that

$$S(i, X, m)(t) = S(j, X, m)(t + T_{i,j}), \quad 1 \leq i, j \leq N_L, \; X \in \mathcal{X}.$$

Now our second claim follows directly, since the purely labeled X^{p^i} are directly proportional to $S(i, X, n_X)$, and thus,

$$X^{p^i}(t) = \frac{S(i,X,n_X)(t)}{n_X} = \frac{S(j,X,n_X)(t+T_{i,j})}{n_X} = X^{p^j}(t)(t + T_{i,j}).$$

□

This proof is theoretically applicable not only to metabolic networks but also to other systems, as long as they satisfy the assumptions made.

Assumption 1 can be made, as in our case, the labels are chemically similar alkyne-fatty acids themselves, and therefore, we can make the reasonable assumption that they do not influence what kind of reaction can or cannot occur[2]. Furthermore, we choose fatty acids of very similar chain lengths to ensure that the metabolism of those fatty acids is comparable. When using alkyne fatty acids as labels, lipid beta-oxidation has to be considered. Lipid beta-oxidation can change the overall chain length of the alkyne fatty acids. The generic pathways of fatty acid modification will elongate by C_2 units, remove C_2 units, and change the number of double bonds. However, since the resulting products still change in either even or odd chain length or heavy isotopes, we can attribute them as descendants of the unchanged FA[3]. The difference in metabolism of odd and even chain fatty acids is mainly in the beta oxidation, which, in the context of this study, is irrelevant. Thus, in order to keep the dimension of the model as small as possible, we do not model the beta oxidation explicitly. Additionally, we do not model double bonds, chain length, or any further configurations of the fatty acids explicitly. Of course, all three play vital roles in lipid metabolism. However, as the fatty acids we trace are of similar chain length, number of double bonds, and double-bond configuration, we can assume their behavior in the lipid metabolism as a whole to be similar. Our model, therefore, captures a defined subset of lipid metabolic processes rather than the complete lipid metabolism.

Modeling these additional structural variables would pose the inevitable problem of dimensionality of the resulting mathematical model and the necessary time for simulations.

Regarding Assumption 2, we deem it justifiable, as we apply media with identical composition with the sole exception of the fatty acid being a different one. Therefore, the two points in Assumption 2 are satisfied as long as the uptake of fatty acids is equal for the differing labels, which we already assumed with Assumption 1. For the synthetic data generation, we consider the case of a highly dynamic range of the concentrations and, thus, a relatively large percentage of labeled vs. unlabeled metabolites. In less dynamic pools, these percentages can be lower, but the proof and the computational method presented here are independent of this.

Feasibility of Experimental Setup in Bulk Setting

In order to demonstrate the general feasibility of the experimental setup, we performed an experiment on a bulk sample. Freshly isolated hepatocytes were seeded in 12-well plates at a density of 75000 cells per well in Williams Medium supplemented with 10% FCS. For labeling, cells were incubated sequentially with medium supplemented with either unlabeled palmitic acid, FA 16:0;Y, FA 16:0[^{13}C$_9$];Y or FA 17:0;Y, each for 30 min at $100 \mu M$ concentration. After the last incubation, the medium was removed, the cells were washed with PBS, and subsequently lysed and processed for mass spectrometry. Identification and quantification of labeled species were done using the LipidXplorer software. For details on the experimental setup and the analysis, we refer to Thiele et al.[4]. All corresponding data are available on GitHub and Zenodo.

A detailed view into the individual lipids (Figure S5) reveals that we identify 342 lipids, of which 235 are TAG, 55 are DAG, and 52 are PC. These numbers are in line with what we would expect. Differentiating within the lipid classes based on the number of labels was in almost all cases possible (Figure S4). An exception to this is $DAG_{(0,1,1,0)}$, i.e. DAG with one FA 16:0;Y and one FA 16:0[^{13}C$_9$];Y (see Figure S1 for the indexing details). We believe this occurs due to an overlapping peak, rendering this specific constellation immeasurable. In addition, no doubly labeled PC species were detected. This finding is consistent with the known lipid composition of hepatocytes, as they favor mixed or polyunsaturated species over dipalmitoyl-PC, which is found rather in the lung[5]. The standard deviation when summed over the labeling ranges anywhere between 10-20% of the mean measurements, which aligns with our ten percent assumption in the simulations.

Overall, the data confirm that the experiment is suitable for generating multiple time points in a single measurement instance. Furthermore, successfully performing the experiment with bulk data demonstrates that indeed, the sensitivity of measurement devices is the major limiting factor. These results indicate that, with ongoing improvements in detection sensitivity, this approach should become feasible for broader applications in the near future.

References

1. Trautz, M. (1916). Das gesetz der reaktionsgeschwindigkeit und der gleichgewichte in gasen. bestätigung der additivität von cv-3/2r. neue bestimmung der integrationskonstanten und der moleküldurchmesser. Zeitschrift für anorganische und allgemeine Chemie *96*, 1–28. doi: 10.1002/zaac.19160960102.

2. Raclot, T. (2003). Selective mobilization of fatty acids from adipose tissue triacylglycerols. Progress in lipid research *42*, 257–288. doi: 10.1016/S0163-7827(02)00066-8.

3. Wunderling, K., Zurkovic, J., Zink, F., Kuerschner, L., and Thiele, C. (2023). Triglyceride cycling enables modification of stored fatty acids. Nature Metabolism *5*, 699–709. doi: 10.1038/s42255-023-00769-z.

4. Thiele, C., Wunderling, K., and Leyendecker, P. (2019). Multiplexed and single cell tracing of lipid metabolism. Nature Methods *16*, 1123–1130. doi: doi.org/10.1038/s41592-019-0593-6.

5. Harayama, T., Eto, M., Shindou, H., Kita, Y., Otsubo, E., Hishikawa, D., Ishii, S., Sakimura, K., Mishina, M., and Shimizu, T. (2014). Lysophospholipid acyltransferases mediate phosphatidylcholine diversification to achieve the physical properties required in vivo. Cell Metabolism *20*, 295–305. URL: https://doi.org/10.1016/j.cmet.2014.05.019. doi: 10.1016/j.cmet.2014.05.019.
